# Selective Pressure Influences Inter‐Biome Dispersal in the Assembly of Saline Microbial Communities

**DOI:** 10.1111/1462-2920.70019

**Published:** 2024-12-19

**Authors:** Mateu Menéndez‐Serra, Joan Cáliz, Xavier Triadó‐Margarit, David Alonso, Emilio O. Casamayor

**Affiliations:** ^1^ Ecology of the Global Microbiome‐Department of Ecology and Complexity Centre of Advanced Studies of Blanes (CEAB), Spanish Research Council (CSIC) Blanes Catalonia Spain; ^2^ Centre for Ancient Environmental Genomics Globe Institute, University of Copenhagen Copenhagen Denmark; ^3^ Theoretical and Computational Ecology Group‐Department of Ecology and Complexity, Centre of Advanced Studies of Blanes (CEAB) Spanish Research Council (CSIC) Blanes Catalonia Spain

**Keywords:** community ecology, dispersal, microbes, selection, stress gradient

## Abstract

Selection and dispersal are the primary processes influencing community assembly at both global and regional scales. Although the effectiveness of dispersal is often examined within the same biome, microscopic organisms demonstrate the capability to colonise and thrive across different biomes. In this study, we evaluated the relationship between (i) aquatic, (ii) sedimentary and (iii) aerial microbial communities, and how local selective pressures influence the potential impact of inter‐biome dispersal, focusing on the salinity gradient stress over time in ephemeral saline lakes. Our taxonomic ordination analyses revealed that the three communities were distinctly segregated yet interconnected by shared populations. Organisms prevalent across the three biomes exhibited cosmopolitan behaviour based on global databases, indicating an inherent ability to cross biome boundaries. Cosmopolitan groups dominated the planktonic community at lower salinities but gradually diminished as salinity increased, resulting in communities dominated by aquatic specialists with more restricted environmental distributions. The aerial community was primarily composed of generalists, although airborne halophiles were also identified, suggesting long‐range dispersal as a source of colonisers in isolated extremophile environments. Our findings contribute to a better understanding of the dynamic interplay between dispersal and selective pressures on community assembly across biomes, highlighting the significance of aerial microbiota in remote colonisation.

## Graphical abstract

Microbial communities in terrestrial, aquatic and aerial biomes are distinct but interconnected. As salinity increases, the contribution of inter‐biome dispersal to local planktonic communities decreases, shifting from generalists to specialists.

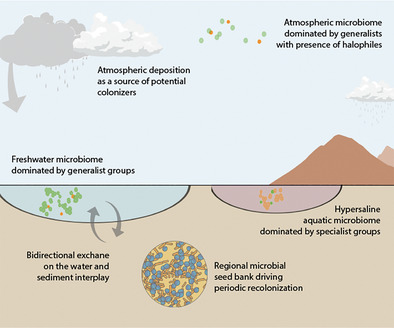

## 1 Introduction

Biological communities are the result of diverse assembly processes simultaneously acting at multiple spatial and temporal scales (Zobel 1997). These processes determine the composition of both regional and local species pools as selected subsets of the common global pool (Barberán, Casamayor, and Fierer 2014, and references therein). The relative contribution of the final community composition of the operating assembly processes (i.e., selection, dispersal, drift and speciation) (Vellend 2010) depends on factors associated with both the organism and the environment characteristics. Global scale (i.e., long‐range) dispersal of organisms is, together with environmental selection, one of the main processes shaping the composition of regional species pools. This process is especially relevant for microbes, whose small size, staggering abundances and high survival skills suggest unlimited dispersal (Becking 1934). Microbial long‐range dispersal events have been observed through atmospheric, oceanic and hydrologic processes (Casamayor et al. 2023; Golan and Pringle 2017; Mayol et al. 2017). The added effect of these events progressively configures regional species pools in different areas, whose settlement will be initially determined by selective processes and later shaped by speciation (Vellend 2010). At a regional scale, microbial traits (e.g., the presence of resistant forms) prevent organisms from extinction under unfavourable conditions forming large microbial biodiversity seed banks (Lennon et al. 2021; Lennon and Jones 2011). Finally, the combined effect of dispersal, drift, environmental filtering and biological interactions determines the composition of local communities as a selected subset of the regional species pool (Leibold et al. 2004; Vellend 2010).

Potential successful dispersal of organisms at global and regional scales has been usually only considered within biomes (e.g., terrestrial, aquatic or aerial biomes). This approach is conceptually valid for most macro‐organisms with physiological specialisation that limits their ability to colonise heterogeneous environments. However, the high physiological and metabolic plasticity of microorganisms (Allison and Martiny 2008) might allow them not only to survive but also to develop successfully within a wide range of biomes. Nonetheless, little is known about the environmental factors mediating dispersal of microorganisms among biomes and their influence on fuelling the successful colonisation of new sink environments.

Atmospheric intercontinental dispersal is considered to have a major role in the exchange of microorganisms among distant habitats, which entails an effect on gene flow and community assembly at a global scale (Womack, Bohannan, and Green 2010), but data are scarce. The atmosphere itself has been traditionally underrepresented in the study of the environmental distribution of microorganisms. The main reasons lie in (i) the conceptual consideration of the atmosphere as a neutral transport medium rather than an environment as itself (Lowe and McPeek 2014) and (ii) the low concentration of microorganisms and the difficulties associated with air sampling that have limited their analysis until very recently (Šantl‐Temkiv et al. 2022). Recent studies have raised doubts about the atmosphere's neutrality as a transport medium between habitats by describing an overrepresentation of groups adapted to high UV radiation and moisture limitation, among other factors (Archer et al. 2019, 2023). The consequence of this environmental filtering is the emergence of distinct phylogenetic patterns (Tignat‐Perrier et al. 2019) which, together with the identification of large‐scale biogeographic patterns (Mayol et al. 2017) and temporal variations (Cáliz et al. 2018; Tignat‐Perrier et al. 2019) points to a well‐defined aerial habitat in which multiple assembly processes might play a prominent role. Although the potential effect of atmospheric microbial communities over sink environments has been initially explored in multiple systems (Archer et al. 2019; Griffin et al. 2007; Mayol et al. 2017; Mescioglu et al. 2019; Sharoni et al. 2015), only a few recent studies have deeply analysed the composition of both sources and sinks to explain the composition of local communities (Archer et al. 2019, 2020).

The role of microbial dispersal between aquatic and terrestrial biomes has also been studied at a regional scale. Terrestrial‐derived microbial colonisers are known to have a strong influence on the diversity and composition of freshwater plankton (Crump, Amaral‐Zettler, and Kling 2012; Monard et al. 2016; Ortiz‐Álvarez et al. 2020; Ruiz‐González, Niño‐García, and del Giorgio 2015). However, most of these studies have mainly focused on unidirectional systems where the flux of colonisation is easily traceable, occurring in most cases exclusively from soils to water masses. Therefore, the community assembly processes occurring in the context of a bidirectional exchange (e.g., as expected for the interphase water‐sediments) remain poorly explored.

In this context, ephemeral saline lakes in continental areas appear as a potentially ideal model system to explore the role of both long‐range airborne and sediment‐derived microbial colonisers on plankton communities. In most cases, these saline inland environments are geographically isolated shallow wetlands surrounded by uplands placed in remote watersheds, being therefore long‐range airborne dispersal the main potential income of halophiles from distant sources. Additionally, the relationship between water and sediment is extremely complex in ephemeral ponds. During desiccation stages, dry sediments act as a seed bank hosting microorganisms which will recolonise the aquatic environment after pond re‐inundation in rain periods (Sisson et al. 2018). We aimed to test the linkages between plankton, sediment and aerial microbial communities and how the potential dispersal of microorganisms among biomes could contribute to shape local communities. We hypothesised that the potential effect of inter‐biome microbial dispersal on the planktonic assembly and the resulting community composition will be variable and strongly modulated by the selective pressures changing along the dynamic local salinity gradient.

## 2 Materials and Methods

### 2.1 Study Sites and Genetic Data Sets

**FIGURE 1:**
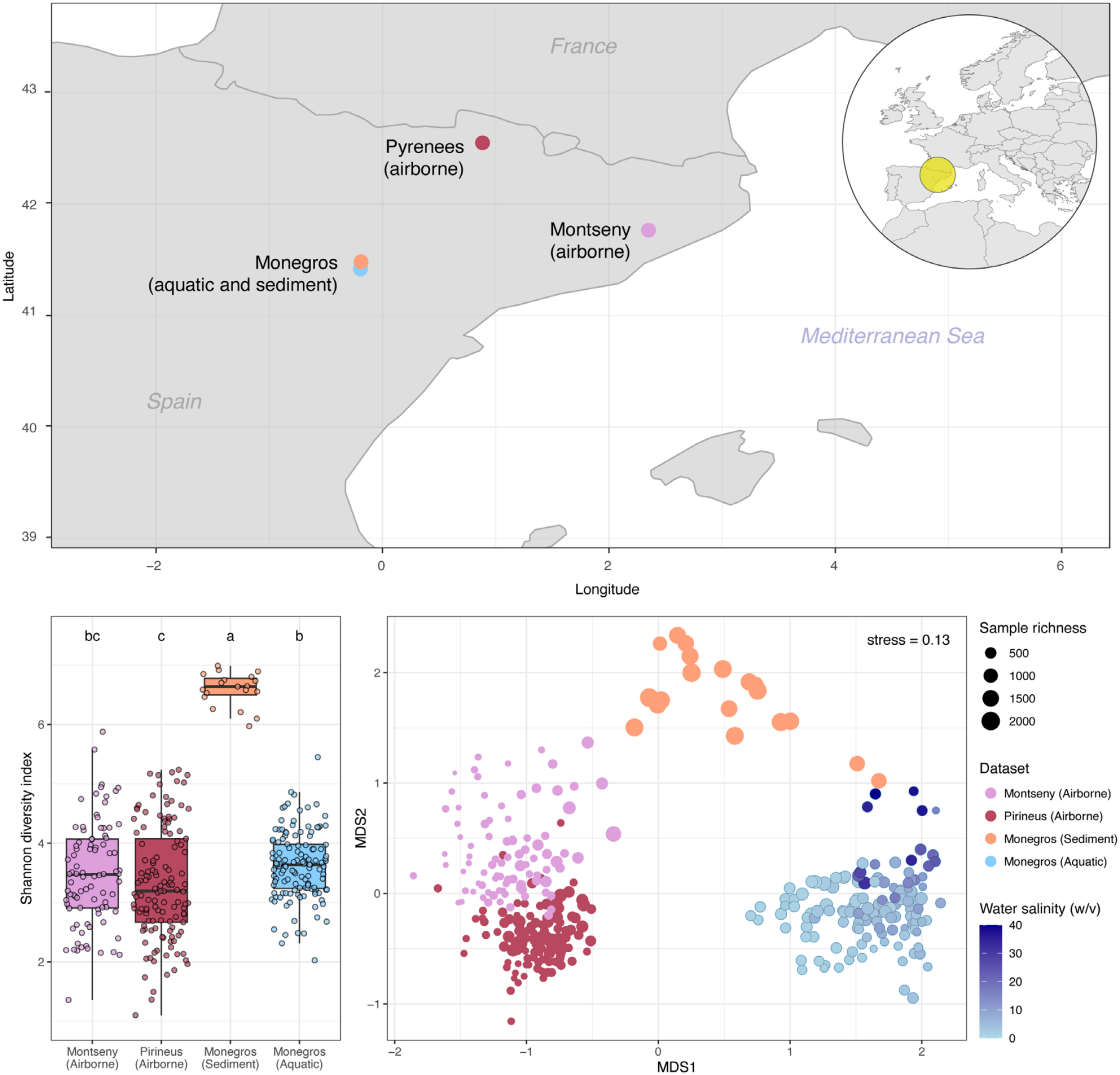
Geographic location of the Monegros, Pyrenees and Montseny sampling sites (top). Shannon diversity index for the microbial composition in each biome (boxplot, bottom left); letters indicate significant differences among biomes according to Tukey's range test after significant ANOVA. Non‐metric multidimensional scaling (NMDS) representation based on pairwise Bray–Curtis dissimilarities among samples (bottom right).

We investigated four 16S rRNA gene data sets from sites separated a few hundred kilometres in NE Spain (Figure 1, upper panel) covering (i) plankton, (ii) sediment, and tropospheric microbiota collected in two mountain areas, (iii) Pyrenees and (iv) Montseny, respectively, to test for potentials links. The aquatic and sedimentary data were obtained from the Monegros desert lacustrine system (41°42′N, 0°20′W). The microbiota of the Monegros wetlands has been previously described as this is one of the largest sets of inland saline lakes in Europe, whose environmental characteristics and accessibility make it of special interest for the study of a wide range of ecological aspects and temporal surveys (Casamayor, Triadó‐Margarit, and Castañeda 2013; Menéndez‐Serra et al. 2019, 2020; Menéndez‐Serra, Triadó‐Margarit, and Casamayor 2021). The aquatic data set contained 135 samples from 14 saline lakes taken over three consecutive years (2012–2014) and captured a high environmental variability, mostly represented by wide salinity fluctuations associated with the hydrological cycle dynamics (Menéndez‐Serra et al. 2020; Menéndez‐Serra, Triadó‐Margarit, and Casamayor 2021). The sediment data set consisted of 19 samples available from five saline lakes, collected in multiple campaigns between 2010 and 2014 (Menéndez‐Serra et al. 2019). Sediment dry samples were sampled within the inner margin of the ponds during a desiccation stage, being therefore influenced by water masses during pond inundation periods. The atmospheric microbiota was temporally collected from wet atmospheric precipitation in two long‐term mountain stations in NE Spain. The Pyrenees data set (*n* = 129, covering the period 2007–2014) was collected at the High Mountain LTER‐AT site, within the protected area of the Aigüestortes i Estany de Sant Maurici National Park in the Pyrenees (42°33′N 0°53 E, c.a. 1700 m a.s.l.) (Cáliz et al. 2018). The Montseny data set (*n* = 89, covering the period 1987–2014) was collected at La Castanya station within the Montseny massif (41°46′N, 2°21′E, c.a. 700 m a.s.l) (Avila and Rodà 2023; Cáliz et al. 2025). The high‐altitude and deposition‐based sampling strategy preferentially captures a representation of the regional‐to‐global atmospheric microbiota rather than local airborne microorganisms (Casamayor et al. 2023). The tropospheric aerial microbiota experience long‐distance intercontinental dispersal travelling in the free troposphere above the atmospheric boundary layer (dominated by turbulent and local winds) until scavenging by precipitation (rain and snow) and gravity (dry deposition) facilitates the deposition of microbes into new habitats, covering extensive areas.

The analysis of all molecular data sets was carried out using the same primers set (V4 region of the 16S rRNA gene, 515f‐806r; Caporaso et al. 2010) and sequencing platform (Illumina 2 × 250 MiSeq). Raw sequences from the four data sets were processed together following the UPARSE pipeline (Edgar and Flyvbjerg 2015). Sequences were merged and quality filtered (expected error of 0.25). After de‐noising and chimera filtering, the UNOISE algorithm (Edgar 2013) defined Operational Taxonomic Units at 100% identity, that is, zero‐radius OTUs (zOTUs). The taxonomic assignment was carried out with SINA aligner v.1.2.11 (Pruesse, Peplies, and Glöckner 2012) and the SILVA 132 reference database (Quast et al. 2013). zOTUs were filtered by their alignment quality score (> 80%), and those classified as mitochondria and chloroplasts were removed. Only samples with > 9.000 reads were retained, and the resulting table consisted of 22.261 zOTUs distributed in 368 samples. All gene sequence data sets have been separately deposited to the NCBI Sequence Read Archive and are available through BioProject record IDs PRJNA429605 (water samples), PRJNA420947 (sediment samples), PRJEB14358 (aerial samples from Pyrenees mountains) and PRJNA1004844 (aerial samples from Montseny mountain), respectively.

### 2.2 Statistical Analyses

Statistical analyses were run in R environment (http://www.r‐project.org/). Ecological parameters, such as richness and the Shannon–Weaver diversity index, were calculated using *vegan* package version 2.4‐5 (Oksanen et al. 2016) after rarefying the reads of all samples to the minimum number of reads per sample. For community‐related analysis, rarefaction at the same depth was repeated 100 times to avoid the loss of less abundant zOTUs and the resulting tables were unified on an average rarefied zOTU table. Spearman's rank‐order correlation coefficient was carried out to test monotonic associations between numeric variables. Differences among group means were tested using Tukey's range test, function *HSD.test* within *agricolae* package version 1.2‐8, after significant one‐way ANOVA. Differences in the microbial composition among aquatic, sedimentary and aerial samples were assessed by non‐metric multidimensional scaling (NMDS) ordinations and permutational multivariate analysis of variance (PERMANOVA) using distance matrices based on Bray–Curtis dissimilarities after Hellinger standardisation (Legendre and Gallagher 2001). The distribution and relative abundance of the most abundant zOTUs were shown as a heat map in which zOTUs were clustered based on their mean abundance within each biome; function *hclust* within the *stats* package version 3.6.2. Abundant zOTUs were defined as those with a mean abundance within the biome ≥ s 0.25%, resulting on 132 zOTUs representing a mean relative abundance per sample of 0.56 (SD ± 0.24).

### 2.3 Environmental Ontology Sequences Annotation (*EnvO Terms*)

We use *seqenv* (Sinclair et al. 2016) to link gene sequences to potential sources based on the reported habitat for similar sequences available in public repositories. For a given zOTU sequence, *seqenv* performs a BLAST search against the NCBI nucleotide database (last compatible version from 2015) to detect sequences with high identity and applies a text‐mining protocol to extract environmental ontology terms from the data associated with those sequences. Environmental ontology (EnvO) terms are based on a concise, structured and controlled vocabulary which helps to define environmental entities of all kinds (Buttigieg et al. 2013). Despite the potential intrinsic limitations associated with a database‐dependent method, the information extracted from this approach can still provide useful insights into the potential distribution of taxa at a global scale. The *seqenv* pipeline (version 1.3.0) was run over the predicted zOTUs sequences with a minimum identity threshold of 99%. As each zOTU might be linked to several *EnvO* terms, the relative contribution of each term was determined by the proportion of matched sequences reported from each source. This allowed us to assign to each zOTU a richness and diversity of sources according to the different environments where a given zOTU was previously detected, which were used as a proxy for habitat specificity or generalism at the global scale (i.e., lower or higher richness and diversity of sources, respectively).

### 2.4 Specificity Index

We applied the specificity index reported by Mariadassou, Pichon, and Ebert (2015) to determine the specificity of each zOTU to each of the studied biomes. The specificity index is based on the *IndVal* index (Dufrene and Legendre 1997) but keeps the specificity value of each zOTU within all groups of samples, not only its maximum. We selected zOTUs detected within the aquatic biome to test their specificity to each salinity range and to their potential source compartments (i.e., airborne and sediment). Five salt ranges were defined as follows: slightly brackish (< 1.5% w/v), brackish (1.5%–2.9%), marine (3%–4.9%), hypersaline (5%–14.9%) and highly hypersaline (> 15%) according to previously described thresholds based on physicochemical and biological factors (Edgerton and Brimblecombe 1981; Imhoff 2001; Kushner 1978; Por 1972; Rodriguez‐Valera 1993). The specificity index was represented against the mean abundance of each zOTU within each sample to assess the potential link between specificity and dominance (Mariadassou, Pichon, and Ebert 2015). The significance of the observed trends was tested by permutations to discard potential artefacts. For this, the same index was calculated for each zOTU after randomly assigning each sample to a given category while keeping constant the number of samples of each group. This process was repeated 999 times and the mean index value was computed for each zOTU and sample group. Then, both the observed data and the mean values of the permutation test were fitted to a linear model and their slopes were statistically compared (function *lstrends* from *lsmeans* package) to estimate potential differences between trends.

Overall, we used a combination of two complementary approaches: (1) the joint analysis among plankton, sediment, and aerial communities and (2) the use of a database source tracking approach to determine potential environmental sources based on gene sequence identities. Using this combination, we tested (i) the linkage between the microbial communities inhabiting all three biomes, (ii) the variable contribution of airborne and sediment colonisers into the planktonic assemblage along the hydrological cycle and (iii) the distribution of habitat generalists and specialist in the plankton of the ephemeral saline ponds studied.

## 3 Results

### 3.1 Analyses of the Community Composition Across Biomes

**FIGURE 2:**
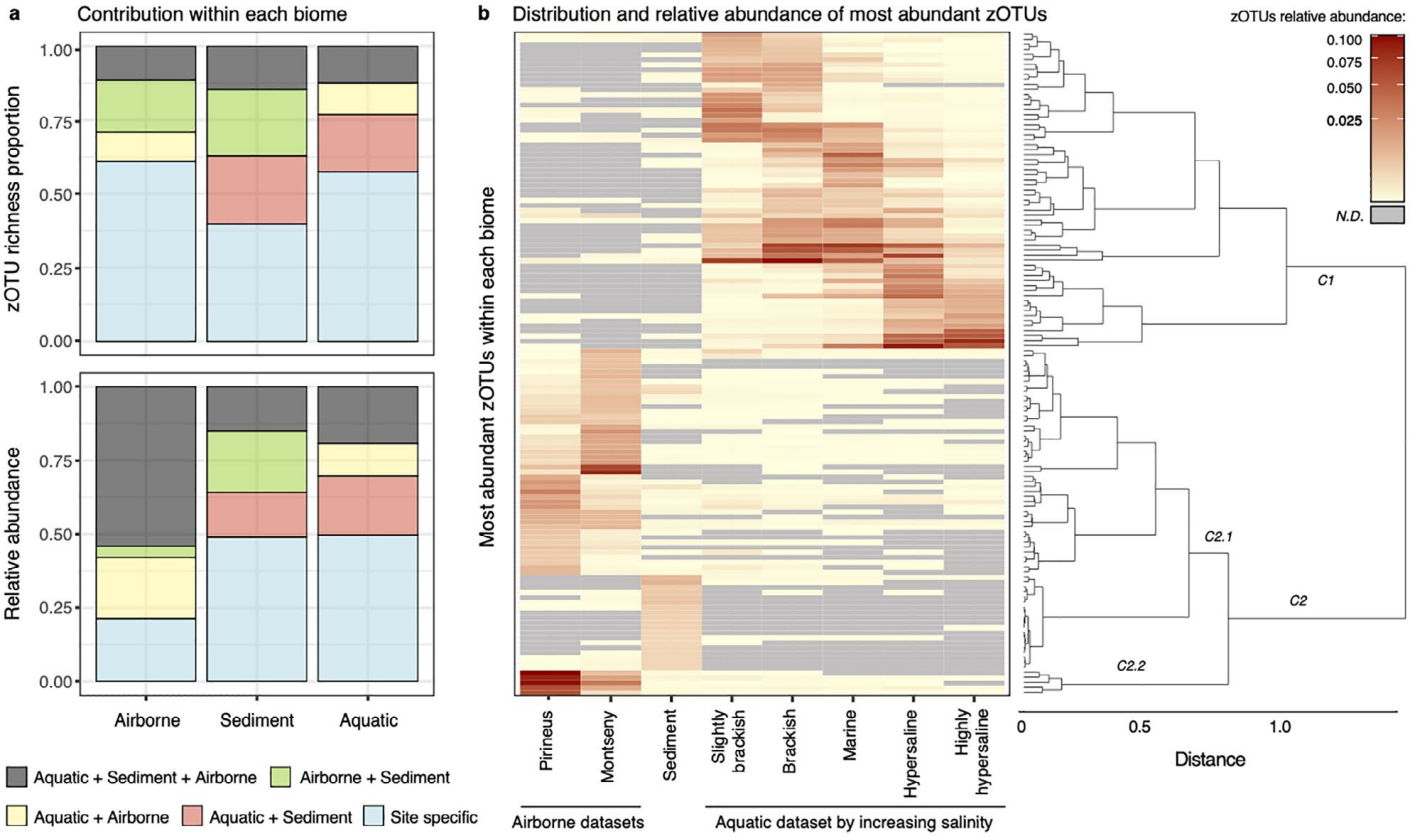
(a) Proportion and relative abundance of zOTUs assigned following detection in the different biomes. (b) Distribution and relative abundance of the most abundant zOTUs within each biome and salinity range in the case of plankton. zOTUs clustering is based on their relative abundances in each compartment. ND, non‐detected.

The composition of the microbial communities of the three biomes showed significant differences (Bray–Curtis dissimilarity, ANOSIM statistic R: 0.96, *p* value < 0.001) (Figure 1, bottom left panel). Although sediment samples constituted the data set most limited in size, it showed a significantly (*p* value < 0.001) much higher zOTU richness (one‐way ANOVA, *F* = 95.58, and see also dots sizes in Figure 1, bottom right panel) and Shannon diversity (one‐way ANOVA, *F* = 456.1) than the other two biomes. We noticed, however, that a subset of 1261 zOTUs (5.7% of total zOTUs) was shared among all three biomes (Figure S1). These cosmopolitan zOTUs had a similar richness proportion in all biomes (c.a. 20%) but a much higher relative abundance (c.a. 55%) in aerial samples than in sediments and plankton (c.a. 20%) (Figure 2a). We also identified specific populations (i.e., those only detected in one of the biomes), for aquatic (5852 zOTUs), aerial (6700 zOTUs) and sedimentary (3430 zOTUs) biomes, reaching ~60% of richness in airborne and plankton communities and ~40% in sediments, respectively. Biome‐specific zOTUs reached < 25% of relative abundance in airborne samples, and ~50% in plankton and sediment, respectively.

The clustering analysis based on the distribution and relative abundance of the most abundant zOTUs showed two main clusters (Figure 2b): Cluster C1 for plankton grouped according to their salinity preferences, and Cluster C2 for zOTUs with maximum abundances in airborne and sediment samples, respectively. The subcluster C2.2 included five zOTUs assigned to *Methylobacterium* (zOTU1), *Sphingomonas* (zOTU2, zOTU5 and zOTU6) and *Massilia* (zOTU3) which had maximum abundances in airborne samples in agreement with the previous description of atmospheric deposition (Woo and Yamamoto 2020) and were widespread, at lower abundances, across all biomes. This analysis evidenced that most zOTUs showing intermediate‐to‐high abundances in a given biome were residual or absent in the other two biomes, that is, even widespread zOTUs showed a dominant affiliation to one of the tested biomes (Figure S2). Furthermore, the analysis highlighted that most of the zOTUs primarily associated with the airborne biome (according to mean abundance and occurrence) were also detected in aquatic and/or sediment samples. However, this observation did not hold true for plankton and sediment, where a remarkable proportion of the dominant zOTUs were biome‐specific (Figures 2 and S3), which might suggest a potential directionality for the inter‐biome colonisation.

### 3.2 Changes in Composition and Environmental Sources Along the Salt Gradient

**FIGURE 3:**
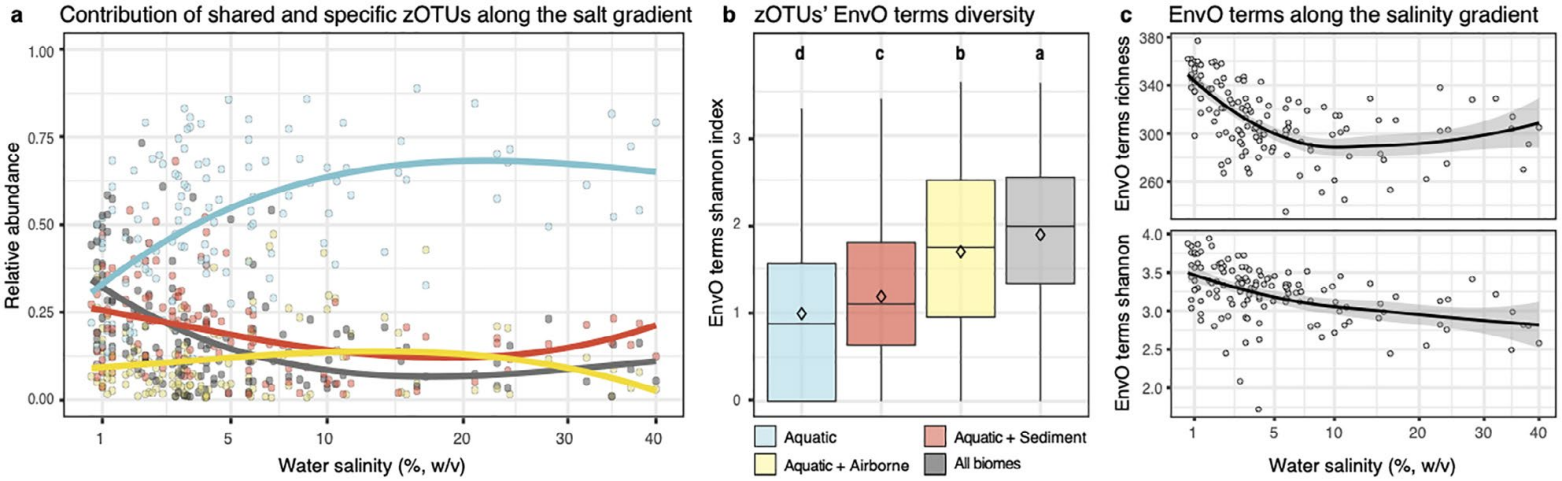
(a) Relative abundance along the salt gradient of the plankton zOTUs detected in the different biomes. (b) Boxplot representation of zOTUs' EnvO terms Shannon diversity index. (c) Aquatic communities mean EnvO terms richness and Shannon diversity index along the salinity gradient.

The contribution of aquatic‐specific populations significantly increased along the salinity gradient, both in zOTU proportion (Spearman's *ρ* = 0.79, *p* value < 0.001) and in relative abundance (Spearman's *ρ* = 0.52, *p* value < 0.001, Figures 3a and S4), whereas those zOTUs shared with airborne and/or sediment communities decreased. Most of this transition was observed at salinities < 10%, where the relative abundance of aquatic‐specific zOTUs increased from ~30% to ~65% and reached stabilisation. Simultaneously, widespread zOTUs decreased from ~37% to ~11%, those present in water and sediment samples decreased from ~18% to ~12% and those detected in water and airborne decreased from ~15% to ~13%. Beyond the 10% of salts, the assembly was mostly dominated by halophile groups only detected in aquatic samples such as Nanohaloarchaea and Woesearchaea and the bacteria *Salinivibrio*, *Spiribacter*, *Halonotius*, *Halothioacillus* and *Salinibacter*. Interestingly, we also unveiled halophiles abundant in the highly hypersaline samples (> 15% w/v) that were also present in air and/or sediment. For instance, we found unclassified Haloferaceae, *Halomonas* and *Marivita*, in the aerobiome; and *Halobacteria*, *Halanaerobiia*, *Halomonas* and *Marivita*, in sediments (Figure S5). In all cases, widespread halophiles presented low abundances in both airborne (mean relative abundance < 0.45%) and sediment (mean relative abundance < 0.16%) samples compared to their optimal salinity range in the aquatic biome (up to 60% on specific samples) (Figure S6).

We also studied the changes in the diversity and richness of environments where each microbe had been previously detected. The diversity of sources showed significant differences according to the global biomes where a zOTU had been detected (one‐way ANOVA, *F* = 295.1, *p* value < 0.001). Interestingly, plankton‐specific zOTUs showed the lowest diversity of global sources, whereas zOTUs shared in all three biomes showed the highest (Figure 3b). We also observed a significant decrease in both the mean diversity (Spearman's *ρ* = −0.52, *p* value < 0.001) and richness (Spearman's *ρ* = −0.59, *p* value < 0.001) of predicted sources along the salinity gradient (Figure 3c). A sharp reduction of both indicators occurred up to a 10% salinity threshold (Spearman's *ρ* = −0.65 and −0.40 for richness and diversity, respectively, *p* value < 0.001), and non‐significant decrease was further observed in the range 10%–40% salinity.

### 3.3 Microbial Specificity and Generalism Along the Salt Gradient

**FIGURE 4:**
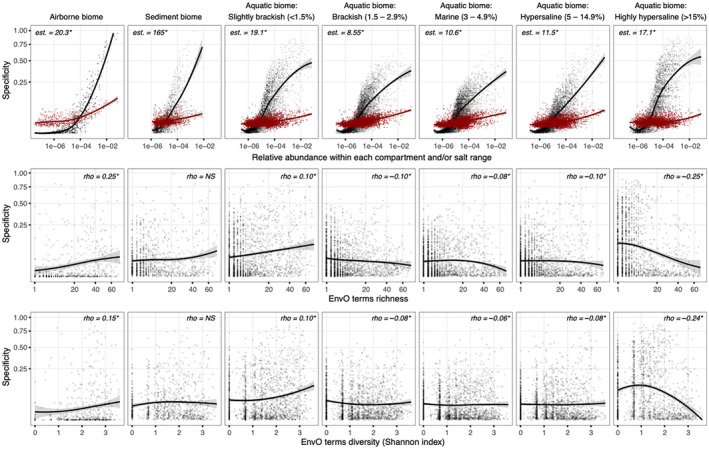
Positive relationship between specificity index and local abundance for each zOTU and tested group (i.e., airborne, sediment and samples classified within each salinity range on the aquatic biome). Only zOTUs detected within aquatic samples were considered. Red dots and trends represent the expected relationship under the null distribution when samples are randomly assigned to each group of samples. Differences between observations and permutations test were approximated by slope comparisons after fitting to a linear model (top panel). Specificity index and salinity range represented against the richness and diversity (richness in the middle, and Shannon index in the bottom panel) of sources (EnvO terms) for each zOTUs. Significance codes: *p value < 0.001. NS, non‐significant correlation.

The specificity index calculated for each zOTU and sample group (*airborne*, *sediment* and *plankton* for the different salinity ranges) showed a positive correlation with zOTUs' local abundance in all cases (Figure 4, top panels). This finding indicates that the most abundant zOTUs for a given sample group had the highest specificity, that is, most samples were dominated by specific taxa rather than by widespread organisms. Slope comparison against the null model results, after the adjustment to linear models, showed significant differences in all the cases. For plankton, maximum differences between observations and permutation tests were noticed at both ends of the salt gradient. These differences suggest a higher dominance of what we defined as local (within the scope of the explored aquatic data set) specialists at both the lowest and the highest salinities, that is, taxa dominating at both ends of the gradient did not successfully develop outside their preferred environmental niche. Conversely, taxa dominating at intermediate salinities might have had wider niches and therefore prevailing across wider salt ranges.

We then plotted the specificity index against the information provided by the *seqenv* pipeline (Figure 4, middle and bottom panels for predicted sources' richness and diversity, respectively) to test whether taxa exhibiting local specificity within the assessed compartments were either cosmopolitan or specialists based on global databases information. Within the airborne biome, local specificity showed a positive and significant correlation with both richness (Spearman's *ρ* = 0.25, *p* value < 0.001) and diversity (Spearman's *ρ* = 0.15, *p* value < 0.001) of sources, that is, dominant microbes had a wider distribution of environmental sources and were therefore defined as global generalists. A similar trend was also observed at the lowest salinity range within the aquatic biome (Spearman's *ρ* = 0.10, *p* value < 0.001). However, the trend changed as salinity increased, shifting from positive to negative correlations between local specificity and both richness and diversity of environmental sources (Spearman's *ρ* = −0.25 and −0.24, respectively, *p* value < 0.001, at the most saline end), leading to a predominance of specialist at the local and the global scales.

## 4 Discussion

**FIGURE 5:**
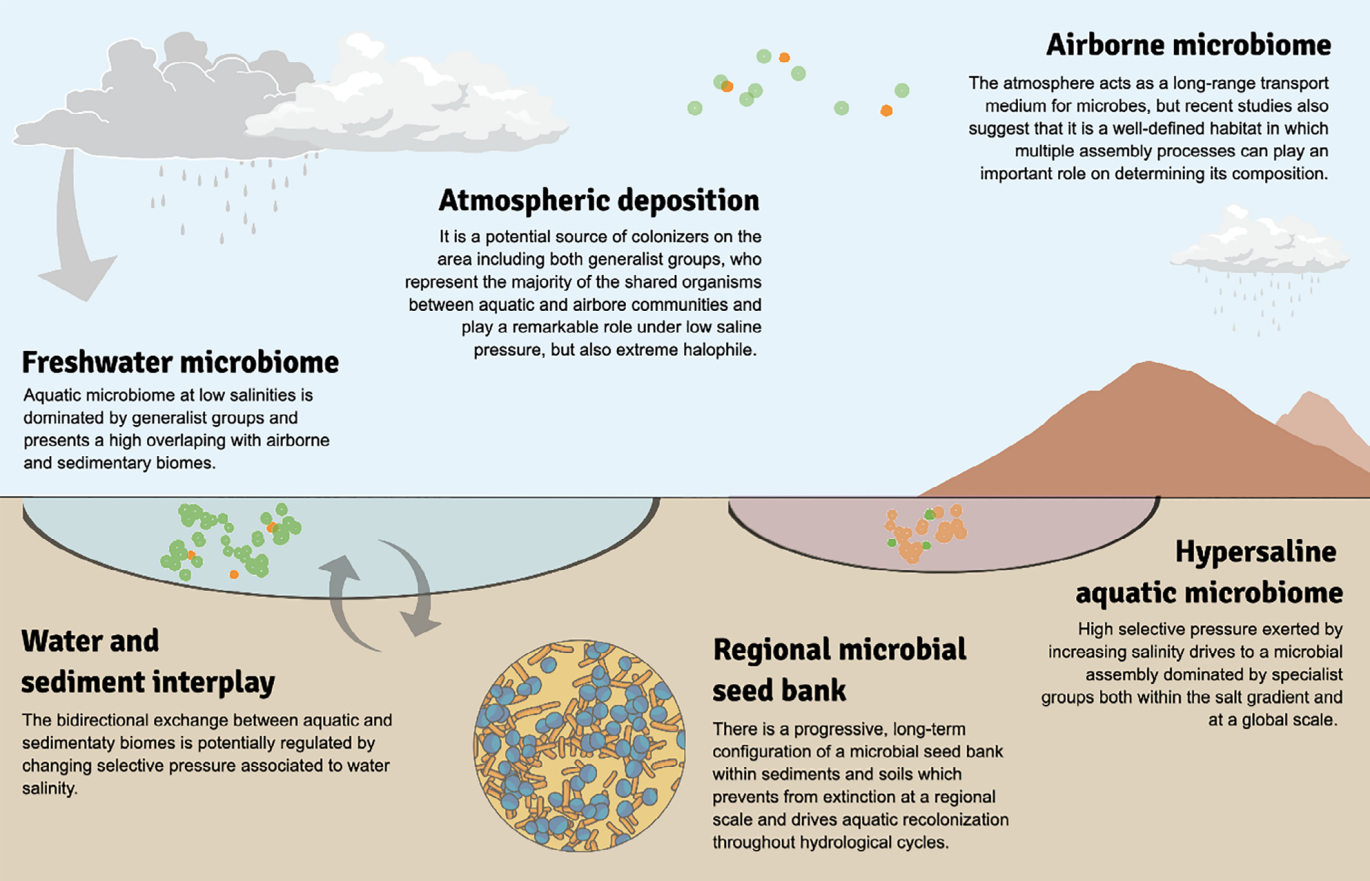
Conceptual framework representing the multiple compartments potentially taking part in the assembly of local aquatic communities in the Monegros area and the potential dynamic interactions among them.

The assembly of biological communities is known to be extremely complex, and a product of multiple assembly processes acting simultaneously at variable spatial and temporal scales (Vellend 2010). The complexity of this process is higher for tiny organisms, since their successful dispersal can occur across biome borders (Crump, Amaral‐Zettler, and Kling 2012; Ruiz‐González, Niño‐García, and del Giorgio 2015), than for macroorganisms. However, the factors modulating inter‐biome dispersal and potential effects on sinking communities are poorly known. Our results support the idea that air, dry sediments and plankton are well‐differentiated but not completely impermeable compartments. This reinforces the need for an inter‐biome perspective to achieve a comprehensive understanding of the local community assembly. Having this inclusive perspective in mind, we assessed the complex role of allochthonous colonisers in the plankton of ephemeral saline lakes. We noticed that the less saline (freshwater) plankton was dominated by generalist, that is, widespread organisms defined according to both a global source‐tracking and the conspicuous presence detected across the three biomes studied, suggesting successful seeding by (long‐distance) aerial and local/regional sediment microbes. In turn, increasing salinity exerted a selective pressure which shaped a transition towards an assembly dominated by planktonic groups exclusively detected in water and with a specialist behaviour according to global databases. Additionally, we split the potential contribution of both the atmosphere and the sediments as microbial seed banks in the progressive configuration of the regional species pool. Overall, we provided novel insights into the assembly of a complex and very dynamic system (see a conceptual schematic representation in Figure 5) in which the balance between inter‐biome dispersal and increasing selective pressure plays a fundamental role in shaping the transition of generalist to specialist taxa along the salt gradient.

### 4.1 Aquatic Communities Assembly, Environmental Filtering and Microbial Specialisation

The microbial overlapping found in the plankton among the three explored biomes, significantly changed along the different salinity thresholds. The highest overlap observed between plankton and the benthic communities (dry sediment) at low salinities was in agreement with previous studies in highly interconnected segments of freshwater hydrographic networks (Crump, Amaral‐Zettler, and Kling 2012; Ruiz‐González, Niño‐García, and del Giorgio 2015) based on a massive inoculum and a progressive selection of better‐adapted taxa. However, even under low selective conditions not all the microorganisms had the capability to cross the environmental barriers and successfully established in multiple compartments, as previously reported (Monard et al. 2016). In line with this, our results also suggest that this ability is intrinsic to each organism and it can be traced back in terms of generalist or specialist behaviour at a global scale following the information provided by public genes databases. Altogether, the joint analysis of multiple biomes and the source‐tracking approach pointed to a dominance of habitat generalists while water salinities remained low. These groups, however, were not able to develop successfully as saline conditions increased throughout the natural hydrological cycle of ephemeral saline lakes.

At higher salinities only aquatic‐specific taxa were selected, which was reflected in a lower diversity and richness of reported sources at a global scale. This habitat specialisation is most likely given due to the increasing specific requirements for life at high salinities (i.e., to cope with salt stress), which progressively reduced organisms' competitivity outside its specific habitat breadths with a threshold up to 10%–15% of salts. Beyond this threshold, the observed transition seemed to reach a plateau and dominant zOTUs behaved as specialists at local and global scales. Substantial functional changes in microbial communities have been reported in the 10%–15% salinity threshold (Gasol et al. 2004). This plateau is also most likely linked to the previously described transition between moderate and extreme halophilic, historically set at 15% of salts (Imhoff 2001; Kushner 1978). The structural osmoadaptation in extreme halophiles limits their adaptation to lower salinity conditions (Oren 2002), showing limited habitat breadths at any studied scale. Above this salinity threshold, salt‐in taxa (i.e., halophilic archaea and bacteria that are characterised by the accumulation of inorganic salts within the intracellular medium and permanent adaptations off the intracellular enzymatic machinery (Galinski and Trüper 1994; Sleator and Hill 2002)) increased its abundance and become a relevant proportion in the assembly (Menéndez‐Serra et al. 2020; Menéndez‐Serra, Triadó‐Margarit, and Casamayor 2021).

### 4.2 Origin and Maintenance of Halophile Groups in Ephemeral Inland Lakes

The high specialisation of the organisms dominating the assembly under hypersaline conditions challenges the hypothesis, potentially valid for freshwater environments, that water microbial communities are mainly recruited from a selected subset of airborne and sediment assemblies. Then, where did these highly specialised organisms come from in the first place? After environmental fluctuations, changes in community compositions might be the result of two different processes: recruitment over time and over space. Recruitment over time is produced when rare or inactive taxa already present in the community increase their abundance due to a fitness increase following the environmental perturbation (Caporaso et al. 2012; Comte et al. 2017a, 2014; Johanna et al. 2012). Conversely, recruitment over space is produced by the selection of specific groups, better adapted to the novel environmental conditions, among the pool of potential colonisers from external sources (Comte et al. 2017a; Crump, Amaral‐Zettler, and Kling 2012; Ruiz‐González, Niño‐García, and del Giorgio 2015). The results presented here together with previous findings in the same study area (Menéndez‐Serra et al. 2023) suggest that both processes could be simultaneously acting following salinity raise in ephemeral endorheic lakes.

Our results supported that taxa recruitment over space can be running at high salinities by the environmental selection from both the pool of potential colonisers from sediments and other local favourable refugia as dry salt areas, but also from intercontinental airborne immigrants since we detected moderate and extreme halophiles present in the aerial samples. These airborne groups included bacteria typical from marine‐like environments such as *Marivita* together with groups owning wider saline breadths such as *Halomonas*. Furthermore, we successfully identified aerial groups with a high specificity to hypersaline environments as representatives of the archaeal family *Haloferacaceae*, which are unable to adapt to low salinities due to their permanent osmoadaptation based in salt‐in strategies (Galinski and Trüper 1994) (Figure S7). The high specificity of these groups requires their dispersal from distant hypersaline environments, supporting the idea that aerial long‐range dispersal might be a source of highly hypersaline organisms, as previously reported for marine counterparts (Comte et al. 2017b). However, many of the extremophiles detected on the lacustrine system were not detected in aerial samples, which might be explained by multiple factors including its presence below detection limits. Although the temporal atmospheric sampling was based on an integrative approach that aimed to capture a representative airborne community pool, the temporal and spatial distances between sampling sites might be masking fine‐scale trends and processes such as the punctual arrival of specific groups. Temporally, the airborne data sets used belong to a long‐term survey on high‐altitude tropospheric aerial microbiota that experience long‐distance intercontinental dispersal travelling in the free troposphere above the atmospheric boundary layer (dominated by turbulent and local winds). Precipitation (rain and snow) and gravity (dry deposition) facilitate the deposition of microbes into new habitats, after being washed out from the free troposphere, covering extensive areas and with high interannual recurrence (Cáliz et al. 2018, 2025). Alternatively, the lack of detection of specialised organisms in airborne communities might imply that these dispersal mechanisms are not robust enough for a recurrent recolonisation of ponds along hydroperiods and that the configuration of the regional species pool by long‐range dispersal events would require longer temporal scales. This would imply the need of considering additional compartments, for example, soils, sediments and other potential favourable refugia, acting as a biodiversity seed bank which would prevent extinction at a regional scale when environmental conditions turn unfavourable by pond desiccation. Likewise, the detection of moderate and extreme halophiles at very low relative abundances within the plankton along the whole gradient also supports recruitment over time as a main mechanism acting in ephemeral salt lakes. The presence of these groups within the water column is most likely the result of their dispersal from multiple sources when ponds re‐inundated at the beginning of the hydrologic cycle, being sediments the main potential source of the permanent physical interaction. In our study, we included the sediments of five lakes covering 4 years approximately and it was more difficult to cover the potential temporal variability of those specific taxa in sediment. This dispersal is most likely constant and not only limited to the initial rewetting stage, being potentially of special relevance due to the physical characteristics of the studied lagoons, which include shallow waters and constant winds in the area (Triadó‐Margarit et al. 2019). However, an accurate estimation of the origin/nature of the inoculum derived from sediments is complex since sediment communities might be at the same time influenced by present or past sinking communities. In fact, although sediment samples constituted the data set most limited in size, their zOTU richness was much higher than the other two biomes (Figure S8). This finding shows that dry sediments act as the largest seed bank and that the water column itself can be identified as a source providing waves of microbial immigrants along successive temporary phases of rewetting‐drying. This temporal dimension draws a much more complex relationship between plankton and sediments than the potentially occurring in previously studied unidirectional systems as the interphase between soil and water (Crump, Amaral‐Zettler, and Kling 2012; Ruiz‐González, Niño‐García, and del Giorgio 2015). In this scenario, sediments not only act as a source of sediment‐specific—or generalist—organisms but have the potential to host aquatic taxa and halophile groups historically developing within the water column and that remain dormant in the sediment during desiccation stages. When ponds re‐inundated, we hypothesise a massive transference from sediments to the water column, which will help to recolonise the aquatic biome as previously reported for vernal pools (Sisson et al. 2018). Nonetheless, our hypothesis apparently showed a notable flaw: many extreme halophiles, although being present on water samples along the whole gradient, were not detected either on airborne communities or in the sediments that are supposed to act as the main seed bank. This could be explained by either alternative refugia as the origin of extreme halophiles, for example, dry salt areas, or because of the extremely low abundance these groups may have within the sediment. To overcome this potential methodological limitation, Ruiz‐González and collaborators (Ruiz‐González et al. 2017) carried out a deeply‐sequencing of soil samples to assess the presence of rare taxa in the seed bank and showed a remarkable increasing detection of the potential ‘reactive’ pool, that is, those groups showing transitions from rare to abundant between communities, up to a sequencing depth of 500,000 reads per sample. Considering the sequencing depth reached in the present study, with an average of approximately 51,000 raw reads per sample for the sediment data set, this limitation in detection could at least partially explain the lack of highly specific halophilic groups found in the sediment. In addition, we cannot rule out the fact that we analysed only the dry sediments of five lakes in the study area and the coverage could have been improved with a more extended spatio‐temporal survey, despite the already very high richness unveiled.

### 4.3 Concluding Remarks

In summary, we have shown that aquatic microbial communities inhabiting ephemeral shallow saline ponds are potentially influenced by both airborne and sediment communities with variable strength along the salinity gradient. The selective pressure exerted by increasing salinity progressively reduced the role of widespread taxa in the planktonic assembly while the contribution of specialist groups increased. Sediments also might play a major role in the microbial assembly at high salinities, potentially acting as seed banks and driving interannual recolonisations not only by habitat generalists but also by highly specialised halophilic groups. Likewise, long‐range aerial dispersal events most likely play a key role in the progressive configuration of the regional species pool, including both highly specialised groups and cosmopolitan taxa. Altogether, our work reinforces the need to broaden the definition of microbial metacommunities by considering the interaction among multiple biomes to improve our current understanding of the complex processes driving the assembly of microbial communities in nature.

## Author Contributions

**Mateu Menéndez‐Serra:** writing – original draft, writing – review and editing, visualization, formal analysis, conceptualization. **Joan Cáliz:** conceptualization, writing – review and editing. **Xavier Triadó‐Margarit:** conceptualization, writing – review and editing. **David Alonso:** writing – review and editing, supervision. **Emilio O. Casamayor:** conceptualization, writing – review and editing, supervision, funding acquisition.

## Conflicts of Interest

The authors declare no conflicts of interest.

## Supporting information


**Figure S1.** Venn diagram representation of the number and the proportion of shared and specific zOTUs on each biome.
**Figure S2.** Pairwise comparison of mean relative abundance of each zOTU within each biome pair.
**Figure S3.** Mean relative abundance (%) and occurrence (%) of each zOTU within each biome. Colour indicates the biomes where each zOTU has been detected.
**Figure S4.** Contribution of airborne and sediment communities to the aquatic assembly along the salinity gradient, based on the Source Tracker approach (Knights et al. 2011) (v.2). For this, airborne and sediment samples were set as a potential source to explain the composition of aquatic samples (sinks).
**Figure S5.** For water samples with salinities ≥ 15%, mean relative abundance of groups which were also detected on airborne, sediment and both biomes.
**Figure S6.** Relative abundance of selected halophile taxa on the airborne and sediment biomes, and across the salinity gradient on the aquatic biome.
**Figure S7.** Proportion of reported sources (EnvO terms) for selected halotolerant and halophilic groups based on the seqenv pipeline results. In agreement with previous literature descriptions, Marivita exhibits a marine‐like distribution, Halomonas present a wider environmental distribution and members of the class Halanaerobiia and the family Haloferacaceae show a clear preference for saline and hypersaline environments.
**Figure S8.** Sample zOTU richness on the studied data sets.

## Data Availability

The data sets generated during and/or analysed during the current study are available in the NCBI Sequence Read Archive through BioProject record IDs PRJNA429605, PRJNA420947, PRJEB14358 and PRJNA1004844.
