# Supplementary material for: Selective Pressure Influences Inter‐Biome Dispersal in the Assembly of Saline Microbial Communities
Source: Environ Microbiol. 2024 Dec 19;26(12):e70019. doi: 10.1111/1462-2920.70019 (PMC11659653; doi:10.1111/1462-2920.70019)
Supplement: Supplementary file 1 — Figure S1. Venn diagram representation of the number and the proportion of shared and specific zOTUs on each biome. Figure S2. Pairwise comparison of mean relative abundance of each zOTU within each biome pair. Figure S3. Mean relative abundance (%) and occurrence (%) of each zOTU within each biome. Colour indicates the biomes where each zOTU has been detected. Figure S4. Contribution of airborne and sediment communities to the aquatic assembly along the salinity gradient, based on the Source Tracker approach (Knights et al. 2011) (v.2). For this, airborne and sediment samples were set as a potential source to explain the composition of aquatic samples (sinks). Figure S5. For water samples with salinities ≥ 15%, mean relative abundance of groups which were also detected on airborne, sediment and both biomes. Figure S6. Relative abundance of selected halophile taxa on the airborne and sediment biomes, and across the salinity gradient on the aquatic biome. Figure S7. Proportion of reported sources (EnvO terms) for selected halotolerant and halophilic groups based on the seqenv pipeline results. In agreement with previous literature descriptions, Marivita exhibits a marine‐like distribution, Halomonas present a wider environmental distribution and members of the class Halanaerobiia and the family Haloferacaceae show a clear preference for saline and hypersaline environments. Figure S8. Sample zOTU richness on the studied data sets. [file EMI-26-e70019-s001.docx]

Supporting information for

**“Selective Pressure Influences Inter-Biome Dispersal in the Assembly of Saline Microbial Communities”**

Mateu Menéndez-Serra, Joan Cáliz, Xavier Triadó-Margarit, David Alonso and Emilio O. Casamayor

**Supplementary Figures**

**
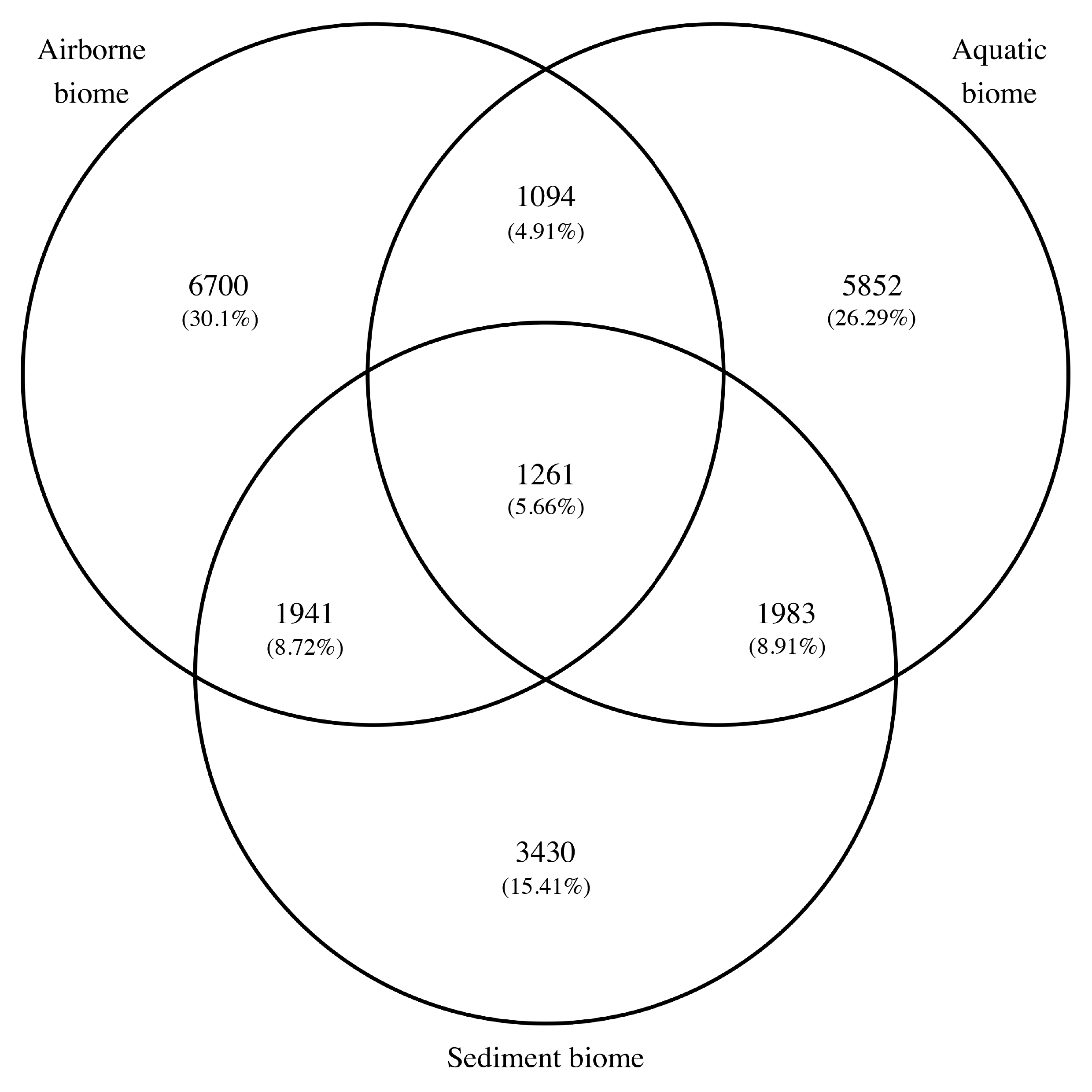
**

***Supplementary Figure S1:*** *Venn diagram representation of the number and the proportion of shared and specific zOTUs on each biome.*


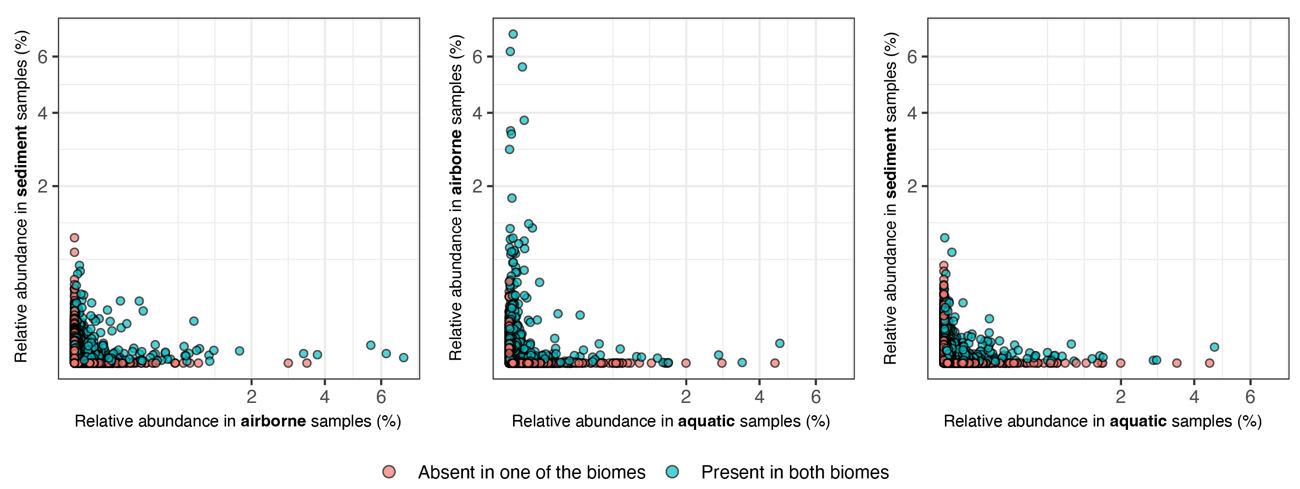


***Supplementary Figure S2:*** *Pairwise comparison of mean relative abundance of each zOTU within each biome pair.*

***Supplementary Figure S3:*** *Mean relative abundance (%) and occurrence (%) of each zOTU within each biome. Color indicates the biomes where each zOTU has been detected.*

**

***Supplementary Figure S4:*** *Contribution of airborne and sediment communities to the aquatic assembly along the salinity gradient, based on the Source Tracker approach (Knights et al., 2011) (v.2). For this, airborne and sediment samples were set as a potential sources to explain the composition of aquatic samples (sinks).*


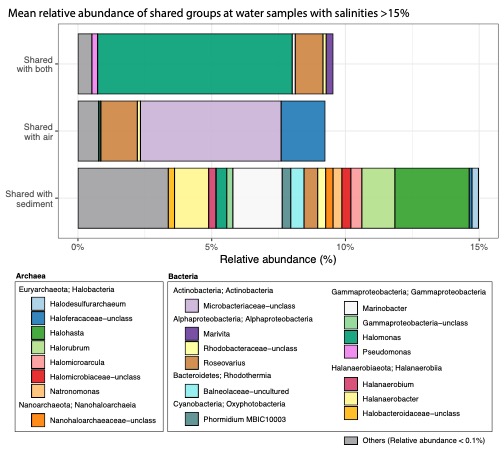


***Supplementary Figure S5:*** *For water samples with salinities >=15%, mean relative abundance of groups which were also detected on airborne, sediment, and both biomes.*

**

***Supplementary Figure S6:*** *Relative abundance of selected halophile taxa on the airborne and sediment biomes, and across the salinity gradient on the aquatic biome.*

***Supplementary Figure S7:*** *Proportion of reported sources (EnvO terms) for selected halotolerant and halophilic groups based on the seqenv pipeline results. In agreement with previous literature descriptions, Marivita exhibits a marine-like distribution, Halomonas present a wider environmental distribution and members of the class Halanaerobiia and the family Haloferacaceae show a clear preference for saline and hypersaline environmets.*

*
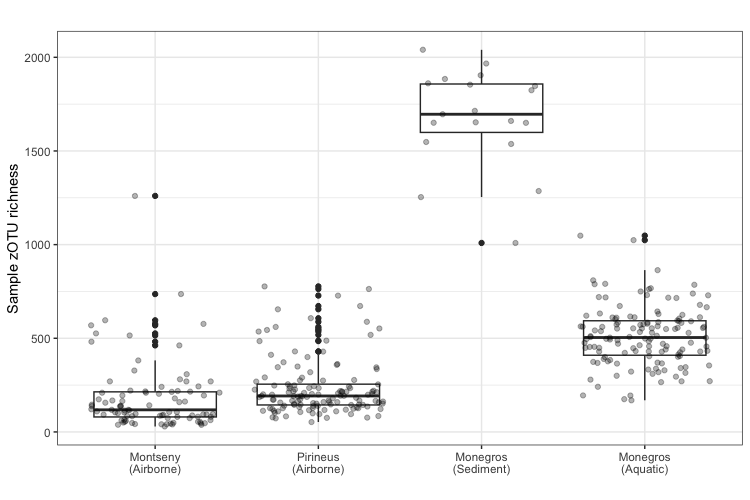
*

***Supplementary Figure S8:*** *Sample zOTU richness on the studied datasets.*
